# Social Insects Dominate Eastern US Temperate Hardwood Forest Macroinvertebrate Communities in Warmer Regions

**DOI:** 10.1371/journal.pone.0075843

**Published:** 2013-10-08

**Authors:** Joshua R. King, Robert J. Warren, Mark A. Bradford

**Affiliations:** 1 Biology Department, University of Central Florida, Orlando, Florida, United States of America; 2 Biology Department, SUNY Buffalo State, Buffalo, New York, United States of America; 3 Yale School of Forestry and Environmental Studies, Yale University, New Haven, Connecticut, United States of America; University of Freiburg, Germany

## Abstract

Earthworms, termites, and ants are common macroinvertebrates in terrestrial environments, although for most ecosystems data on their abundance and biomass is sparse. Quantifying their areal abundance is a critical first step in understanding their functional importance. We intensively sampled dead wood, litter, and soil in eastern US temperate hardwood forests at four sites, which span much of the latitudinal range of this ecosystem, to estimate the abundance and biomass m^−2^ of individuals in macroinvertebrate communities. Macroinvertebrates, other than ants and termites, differed only slightly among sites in total abundance and biomass and they were similar in ordinal composition. Termites and ants were the most abundant macroinvertebrates in dead wood, and ants were the most abundant in litter and soil. Ant abundance and biomass m^−2^ in the southernmost site (Florida) were among the highest values recorded for ants in any ecosystem. Ant and termite biomass and abundance varied greatly across the range, from <1% of the total macroinvertebrate abundance (in the northern sites) to >95% in the southern sites. Our data reveal a pronounced shift to eusocial insect dominance with decreasing latitude in a temperate ecosystem. The extraordinarily high social insect relative abundance outside of the tropics lends support to existing data suggesting that ants, along with termites, are globally the most abundant soil macroinvertebrates, and surpass the majority of other terrestrial animal (vertebrate and invertebrate) groups in biomass m^−2^. Our results provide a foundation for improving our understanding of the functional role of social insects in regulating ecosystem processes in temperate forest.

## Introduction

*To a degree seldom grasped even by entomologists, the modern insect fauna has become predominantly social.* – Bert Hölldobler and Edward O. Wilson, *The Ants.*

The conspicuous presence of social insects in almost all terrestrial ecosystems has captivated the imaginations of biologists, motivating more than a century’s worth of ecological study of ants and termites [1], [2], [3]. Social insects appear most abundant – and most diverse – in the tropics, subtropics, and warm temperate latitudes [1], [2], [4]. Their ecological importance, however, is defined by their influence on nutrient cycling, decomposition, soil engineering, predation upon arthropods, and plant community turnover [5], [6], [7], [8], [9], [10]. Ants and termites are thus described as ecosystem engineers because, along with earthworms, they are typically the only physically large members of the soil invertebrate fauna that are presumed to have sufficient abundance and biomass to influence the formation and maintenance of soil structure and to regulate biological processes across landscapes [6], [7], [9], [11]. Understanding the magnitude of their influence on these ecosystem processes, however, is limited by a lack of data regarding areal abundance (individuals m^−2^) and biomass (grams dry mass m^−2^) estimates [9], [12].

Whereas the engineering effects of earthworms are studied across many systems because their areal abundance is quantified, work on termites as engineers has focused mainly on a few sites in the humid tropics and some African savannas with estimates of areal abundance [2], [9], [13], [14]. Ant engineering effects are, in comparison, little studied as almost nothing is known about their areal abundance [1], [3], [5], [8], [9], [10], [13], [15], [16]. This paucity of data is cited as the reason for omitting ants and termites from syntheses of biogeographical patterns in belowground communities [17], but see also [18]. The shortage of social insect observations probably occurs because most soil fauna studies do not estimate social insect biomass: this requires searching for and collecting whole colonies [19], [20], [21].

The primary focus of our study is to provide areal abundance and biomass estimates of soil macroinvertebrates. That is, invertebrates >2 mm in body width and all ants and termites. This primarily excludes mites and Collembola. We emphasize improving estimates of social insect abundance and biomass across a latitudinal gradient in eastern US temperate hardwood forest [22] to stimulate further investigations into their role in ecosystem processes, paralleling work for salamanders in temperate forest [23]. These forests have a broad geographical range and are important to humans for recreation, carbon storage, timber production and wildlife conservation [24].

Ants and termites are generally warm-loving and numerically dominant in the tropics (and thus described as thermophilic; [1], [9], [13]). So we hypothesized that the range of temperate forests that our sampling covered would show that social insects would increase in areal abundance from northern to southern latitudes, but we expected them to be subdominant to other taxa in these communities, and especially so in northern, cooler regions [2], [25], [26], [27].

## Materials and Methods

### Study Sites

The study was conducted in mid-late August (Connecticut, North Carolina and Georgia sites) and early September (Florida site) of 2011 in four locations spanning ∼12° latitude along the eastern US in second-growth hardwood forests. In Connecticut, the northernmost site, sampling was conducted at Yale Myers Forest in Windham and Tolland Counties (41°57′N 72°07′W). In North Carolina, sampling was conducted at Coweeta Hydrologic Laboratory in the Nantahala Mountain Range in western North Carolina (35°03′N 83°25′W). In Georgia, sampling was conducted at Whitehall Forest, located in the piedmont region of Clarke and Oconee Counties, Georgia (33°53′N 83°21′W). In north Florida, the southernmost site, sampling was conducted at San Felasco Hammock which is in San Felasco State Park, Alachua County, Florida (29°43′N 82°26W). Permits and approval for the work was obtained from the Florida Department of Environmental Protection for permission to work on protected public land at San Felasco Hammock State Park, the US Forest Service and Coweeta LTER for permission to work on the protected public land at the Coweeta Hydrologic Laboratory, the Warnell School of Forestry for permission to work on private land at Whitehall Forest, and the Yale School of Forests for permission to work on private land at Yale Myers Forest.

Yale Myers Forest is managed for timber and is comprised primarily of even-aged northern hardwood species with understory dominated by mountain laurel (*Kalmia latifolia*), gently rolling topography with slopes rarely exceeding 40%, elevation at or below 300 m above sea-level, and temperate climate (mean summer 20°C, winter −4°C, 110 cm annual rainfall [28]). Coweeta Hydrologic Laboratory is a long term site of the USDA Forest Service, Southern Research Station. Elevations range from 675–1592 m and annual climate is temperate (mean summer 21.6°C, winter 1.7°C, 180 cm annual rainfall). Slopes are steep, ranging from 30–100%. Timber was harvested until the 1920 s and currently the forest is largely comprised of even-aged mixed southern hardwood species with a frequently dense understory cover of *Rhododendron* and *Kalmia* species [29]. At Whitehall Forest, elevation ranges from 150–240 m above sea level. The forest is evenly aged (60–70 year old) southern hardwood. Climate is temperate (mean summer 25.6°C, winter 6.7°C, 125 cm annual rainfall). San Felasco Hammock has a topography that is slightly rolling and elevation ranges from approximately 43–52 m above sea level. Climate is southern temperate (mean summer 26.9°C, winter 12.9°C, 132 cm annual rainfall). The forest is secondary growth (selectively logged prior to 1937), even-aged southern hardwood forest with a high diversity of tree and understory species and a well-developed litter layer [30].

Two of the study sites were geographically close and near the center of the latitudinal range (Coweeta and Whitehall) and we recorded the highest average soil temperatures at Coweeta during sampling periods, in spite of its elevation (see Results, below). Thus, to verify that the study sites were representative of a temperate latitudinal gradient we determined the estimated annual temperatures of each site and the above ground productivity of the forest ecosystems present at each site. We used general elevation and latitude lapse rates to generate a temperature index for each site, following Warren and references therein [31]. The relative productivity (annual above ground productivity) of each site was estimated from published values in the literature [32], [33], [34], [35], [36].

### Sampling

At each study site, two 10×10 m plots were established on two north and two south facing slopes (except at YMF, where slopes face East-West), for a total of 8 plots per study site and 32 100 m^2^ plots across all locations. Along each slope, a transect line was randomly placed and one plot was established upslope and another was established downslope, with ∼60 m separating them. Transects were separated by 100 m or more. Macroinvertebrates were sampled in the leaf litter, soil, and coarse woody material (CWM = all dead wood >10 cm dia.). The sampling approach was modified to combine traditional macroinvertebrate sampling (quadrat-based rapid collection of surface material to avoid escapes) while simultaneously sampling whole colonies (ants) or feeding groups (subterranean termites) of social insects in litter, CWM, and soil. Earthworms were captured, however, this study does not properly sample earthworm abundance (c.f. [37]) so earthworm data were not analyzed separately.

**Figure 1:**
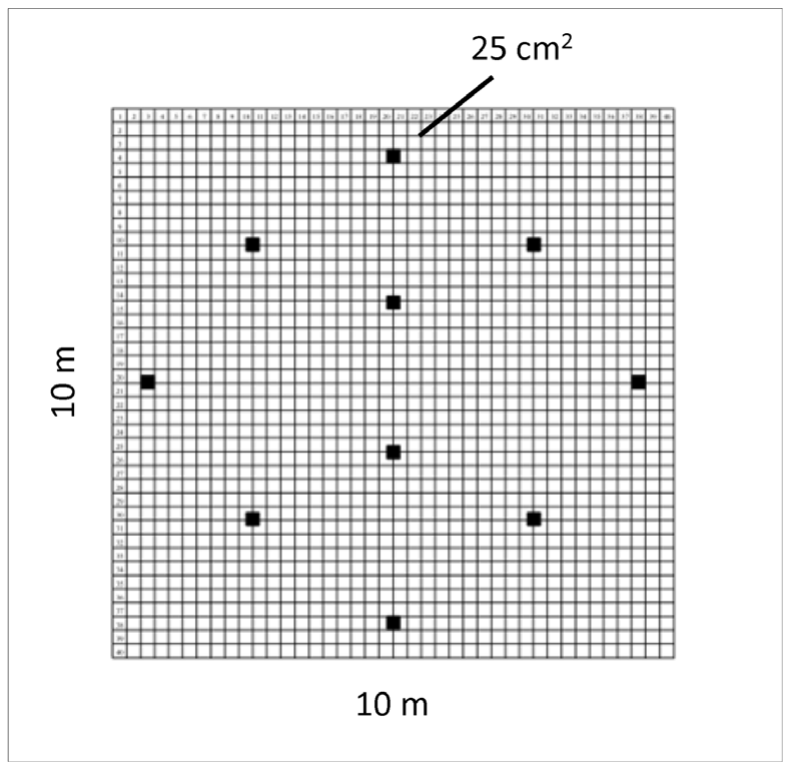
Arrangement of 25 cm^2^ quadrat samples within 100 m^2^ plots.

To sample litter (including fine woody material, FWM = all dead wood <10 cm dia.) and the top ∼5 cm of soil, within each 100 m^2^ plot, ten 25×25 cm quadrats were established in a regularly spaced pattern (Fig. 1). In each quadrat soil temperature was measured at 5 cm depth and volumetric soil moisture (Campbell Hydrosense™) to 12 cm depth. A machete was used to quickly cut (at quadrat edge) any FWM and the surface of the soil within the quadrat. The entirety of the litter sample in the quadrat down to the soil surface was then immediately collected and bagged. The remaining material from the quadrat and the top ∼5–10 cm of soil were then immediately collected using a cordless vacuum (Dewalt™) and bagged. If ants or termites were discovered after vacuuming, all soil to 30 cm depth was excavated to collect the colony. If necessary (i.e. the whole colony was not obviously collected), excavation followed the colony to the depth necessary to get the entire colony (typically no greater than ∼1 m). Within quadrats every effort was made to collect whole colonies. The majority of ant species collected were monodomous (single nest, Table 1). Satellite nests of polydomous species (multiple nest sites per colony) outside quadrats were not collected. Any species observed were noted as collection proceeded if more than one species was present to assure collection of separate, whole colonies.

**Table 1:** Species of ants and termites captured at the four study sites.

Site	Ant species	Termite species
Yale Myers Forest	*Aphaenogaster picea* (Wheeler*)*	
Coweeta Forest	*Aphaenogaster fulva* Roger	*Reticulitermes flavipes(Kollar)*
	*Aphaenogaster picea*	
	*Camponotus chromaiodes* Bolton	
	*Camponotus pennsylvanicus* (De Geer)*	
	*Lasius alienus* (Foerster)*†	
	*Myrmecina americana* Emery	
	*Nylanderia concinna* Trager *†	
	*Nylanderia faisonensis* (Forel)*†	
	*Ponera pennsylvanica* Buckley	
	*Prenolepis imparis* (Say)	
Whitehall Forest	*Amblyopone pallipes* (Haldeman)	*Reticulitermes flavipes*
	*Aphaenogaster picea*	
	*Aphaenogaster rudis* Enzmann	
	*Camponotus castaneus* (Latreille)	
	Nylanderia concinna*†	
	Nylanderia faisonensis*†	
	*Pheidole dentata* MR Smith	
	*Ponera pennsylvanica*	
	*Prenolepis imparis*	
	*Temnothorax curvispinosus* (Mayr)*†	
San Felasco Forest	*Camponotus floridanus* (Buckley)*	*Reticulitermes flavipes*
	*Formica pallidefulva* Latrielle	*Reticulitermes hageni* Banks
	Nylanderia faisonensis*†	
	*Odontomachus brunneus* (Patton)	
	*Pheidole dentata*	
	*Pheidole dentigula* MR Smith	

Ant species noted with an asterisk (*) are polydomous (multiple nests per colony). Ant species noted with a † are polygyne (multiple queens per colony).

After collecting litter, within each 100 m^2^ plot, all CWM was measured along the center axis for length, and at either end for diameter, to estimate the volume (cm^3^). Any CWM falling on the edge of plots was either cut or measured approximately (for pieces too large to cut) and sampled so as to include only CWM inside plots. For CWM small enough to bag, pieces were returned to the lab for sorting. For larger pieces, over a tarp to prevent escapes, every 50 cm of material was inspected and macroinvertebrates were collected.

When ant colonies or termite infestation were encountered in large pieces that could not be returned to the lab (e.g. stumps, large trees), a 15 cm wide piece of wood was collected, at every 50 cm inspection point, for sorting in the lab up to 5 encounters. Using the cordless vacuum, all of the material surrounding the collection was suctioned to assure collection of colonies. Any encounters after the first five were scored as presence and colony size was estimated visually. There were less than ten visual estimates for the entire study. Additionally, five 15 cm wide slices of very large CWM (without visible ants or termites) were taken and the surrounding area vacuumed and sorted in the lab.

All field material was returned to the lab and frozen on the day of collection. All material was later hand sorted after thawing. All woody material was broken apart and all litter material was carefully sorted and inspected. All macroinvertebrates were sorted to Class or Order and termites and ants were sorted to species and counted. All specimens were dried at 65°C prior to weighing. Voucher specimens were taken and currently reside in the University of Central Florida’s insect collection.

### Statistical Analysis

The primary data consisted of the abundance and dry biomass of all macroinvertebrates collected in 100 m^2^ plots and the number of colonies of social insects. Data were converted to m^−2^ and m^−3^ values, which represents an extrapolation of the smaller area sampled. For CWM, total abundance, number of colonies, and biomass could either be reported per unit volume (m^−3^) by dividing by total volume of CWM in 100 m^2^ plots or per unit area (m^−2^) by dividing totals per 100 m^2^ plot by 100. For litter samples, total abundance, number of colonies, and biomass were converted to per unit area (m^−2^) estimates by multiplying totals from all quadrats collected within 100 m^2^ plots by 1.6 [i.e. 10×(0.25 m^2^×0.25 m^2^) ×1.6 = 1 m^2^].

Data were analyzed in SAS version 9 using a mixed-model ANOVA design with number and dry mass of invertebrates as dependent variables and sites (Yale Myers in Connecticut, Coweeta in North Carolina, Whitehall in Georgia, and San Felasco in Florida), sociality (ants and termites versus all other invertebrates), and habitat (litter versus CWM), as classification variables and transect assigned as a random variable. Count data were log_10_+1 transformed and biomass data were log_10_+0.0001 transformed to satisfy normality and homoscedasticity assumptions. As PROC MIXED uses restricted maximum likelihood to estimate unknown covariance parameters, it was necessary to select the best-fitting covariance structure model for the data [38]. The data in all cases were best fit by the most general form possible, an unstructured covariance matrix structure, which was then used to construct the tests for fixed effects.

Approximate Type III *F*-statistics for fixed effects were calculated in PROC MIXED using a general Wald-type quadratic form [38] which we report here as *F*-statistics and associated *P*-values for localities, social and non-social invertebrates, litter and CWM and all two-way interactions. Three-way interactions were not fit because the degrees of freedom were too limited once we accounted for the spatial design of the study. We used an alpha of 0.05 to indicate significance. Inferences for fixed effects in PROC MIXED allows for comparisons across dependent variables while simultaneously accounting for the underlying covariance structure using differences of least squares means. We examined the result of multiple comparisons here as *P*-values after Tukey-Kramer adjustment.

## Results

**Figure 2:**
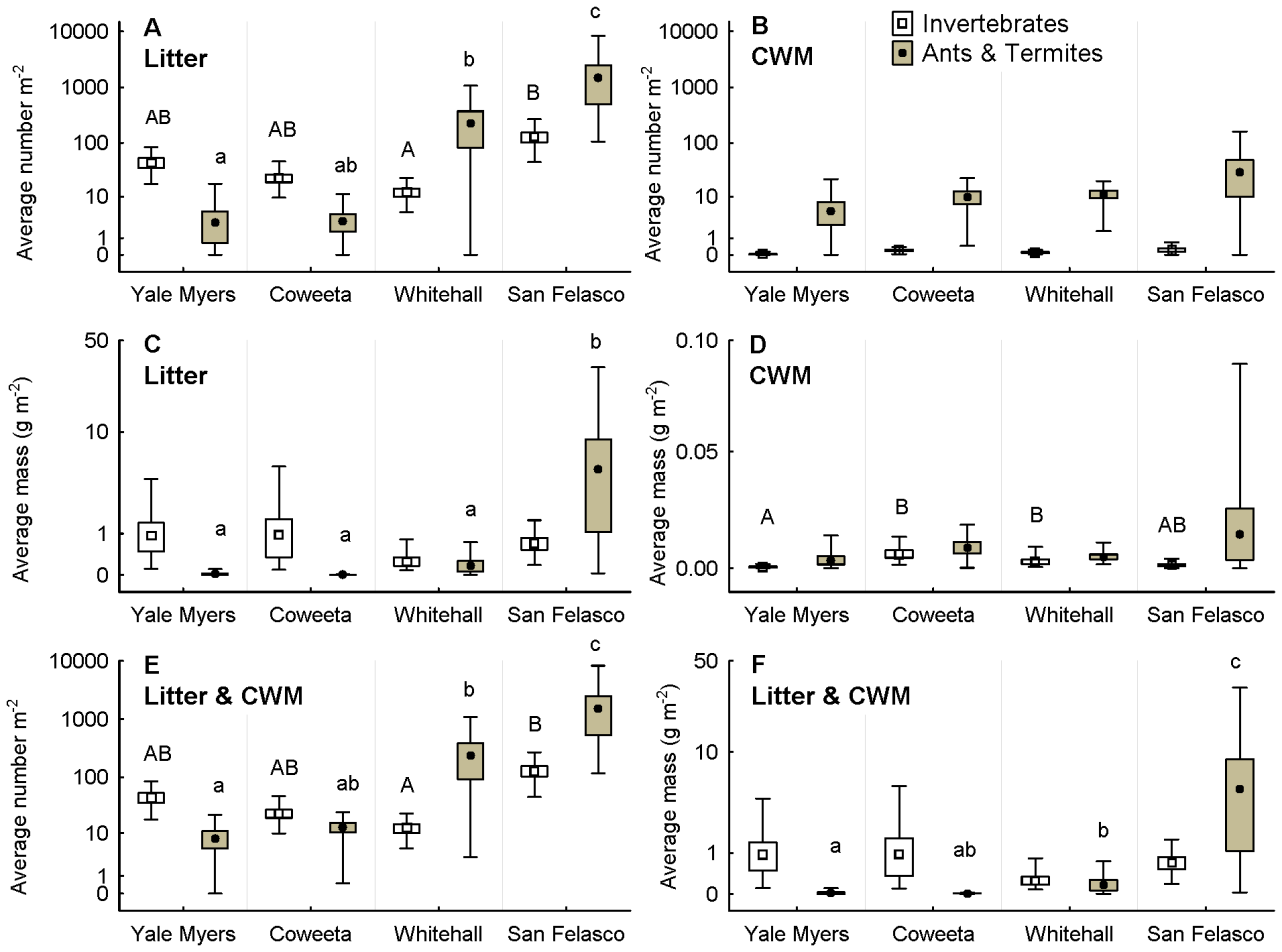
Macroinvertebrate biomass and abundance varied across sites, taxa, and habitats. (A) Average number of non-social invertebrates (not including ants and termites) and social insects (ants and termites) m^−2^ in litter samples and (B) in coarse woody material (CWM) samples. Average ants and termites m^−2^ differed among some sites in litter samples (A) but not in CWM (B). (C) Average dry mass of non-social invertebrates and social insects m^−2^ in all litter samples and (D) in CWM samples. Social insects were more abundant in San Felasco in litter samples (C) while non-social invertebrates only varied among some sites in CWM samples (D). In combined litter and CWM samples, the abundance (E) of both groups varied among sites, while only social insects varied in biomass (F). In both cases, the southern sites had higher numbers and masses of social insects (E and F). Points = mean, bars = +/− SE, and whiskers = range. The Y-axis is log_10_ scaled. Letters above whiskers represent differences revealed through multiple comparisons. Shared letters of the same case (upper vs. lower) indicate no significant differences. Box plots without letters had no significant pairwise difference (Tukey-Kramer adjustment, *P*>0.05).

In the 3,200 m^2^ of forest floor that we surveyed, we found a wide diversity (16 higher taxa) and high abundance (54,417 individuals) of macroinvertebrates (Table 2). Ants (52% of all macroinvertebrates sampled) and termites (45% of all macroinvertebrates) were by far the most abundant organisms overall, becoming increasingly abundant in the southern localities [Georgia (Whitehall) and Florida (San Felasco); Table 2, Fig. 2)]. There were significant differences in the abundance of all invertebrates among sites (*ANOVA*, *F*_3,64_ = 21.1, *P*<0.0001), between social insects and other invertebrates (*ANOVA*, *F*_1,64_ = 6.44, *P* = 0.01), and between microhabitats (CWM vs. litter; *ANOVA*, *F*_1,64_ = 108, *P*<0.0001). There were also significant differences in the biomass of all invertebrates among sites (*ANOVA*, *F*_3,64_ = 12.6, *P*<0.0001), between social insects and other invertebrates (*ANOVA*, *F*_1,64_ = 31.0, *P*<0.0001), and between microhabitats (*ANOVA*, *F*_1,64_ = 137, *P*<0.0001).

**Table 2:** The total abundance and dry mass of macroinvertebrates, from all plots, listed alphabetically by Class or Order.

	Total number	Total dry mass (g)
**Invertebrates**		
Megadrilacea (Earthworms)	7	0.2941
Stylommatophora (Terrestrial Snails)	539	5.3695
Isopoda (Isopods)	33	0.0910
Chilopoda (Centipedes)	81	0.5766
Diplopoda (Millipedes)	216	7.5466
Araneae (Spiders)	96	0.7355
Opiliones (Harvestmen)	9	0.2778
**Insects**		
Blattaria (Roaches)	44	2.0600
Coleoptera (Beetles)	324	3.0643
Diptera (Flies)	5	0.0205
Formicidae (Ants)	28351	35.477
Hemiptera (Bugs)	18	0.2213
Hymenoptera (Sawflies, Wasps, Bees)	8	0.0542
Isoptera (Termites)	24605	14.464
Lepidoptera (Moths and Butterflies)	74	1.6179
Orthoptera (Crickets, Katydids, Grasshoppers)	6	0.3627
Zygentoma (Silverfish)	1	0.0023

Ants are listed separately, as a Family, from other Hymenoptera.

There were significant two-way interactions between the main effects on abundance (*ANOVA*, site×habitat *F*_3,64_ = 14.7, *P*<0.0001; site×sociality *F*_3,64_ = 10.5, *P*<0.0001; habitat×sociality, *F*_1,64_ = 46.7, *P*<0.0001) and dry mass (*ANOVA*, site×habitat *F*_3,64_ = 11.9, *P*<0.0001; site×sociality *F*_3,64_ = 9.43, *P*<0.0001; habitat×sociality, *F*_1,64_ = 68.3, *P*<0.0001) of invertebrates (Fig. 2). These interactions demonstrate the interdependence of latitude, habitat (CWM or litter) and invertebrate type (social insects or other) on the abundance of these organisms. The social×habitat interaction was likely driven by the fact that litter samples produced a much greater abundance of macroinvertebrates than CWM samples, and that this was especially pronounced for non-social taxa (Fig. 2A–D). The site×habitat, and site×social, interactions presumably arose because from north to south there were large differences in macroinvertebrate abundance in litter (Fig. 2A, C) but not CWM (Fig. 2B, D). Social insects were most abundant at the two southern sites [Florida (San Felasco) and Georgia (Whitehall)] irrespective of habitat, but social insects were less abundant in the litter habitat at the two northern sites [North Carolina (Coweeta) and Connecticut (Yale Myers); Fig. 2A].

**Figure 3:**
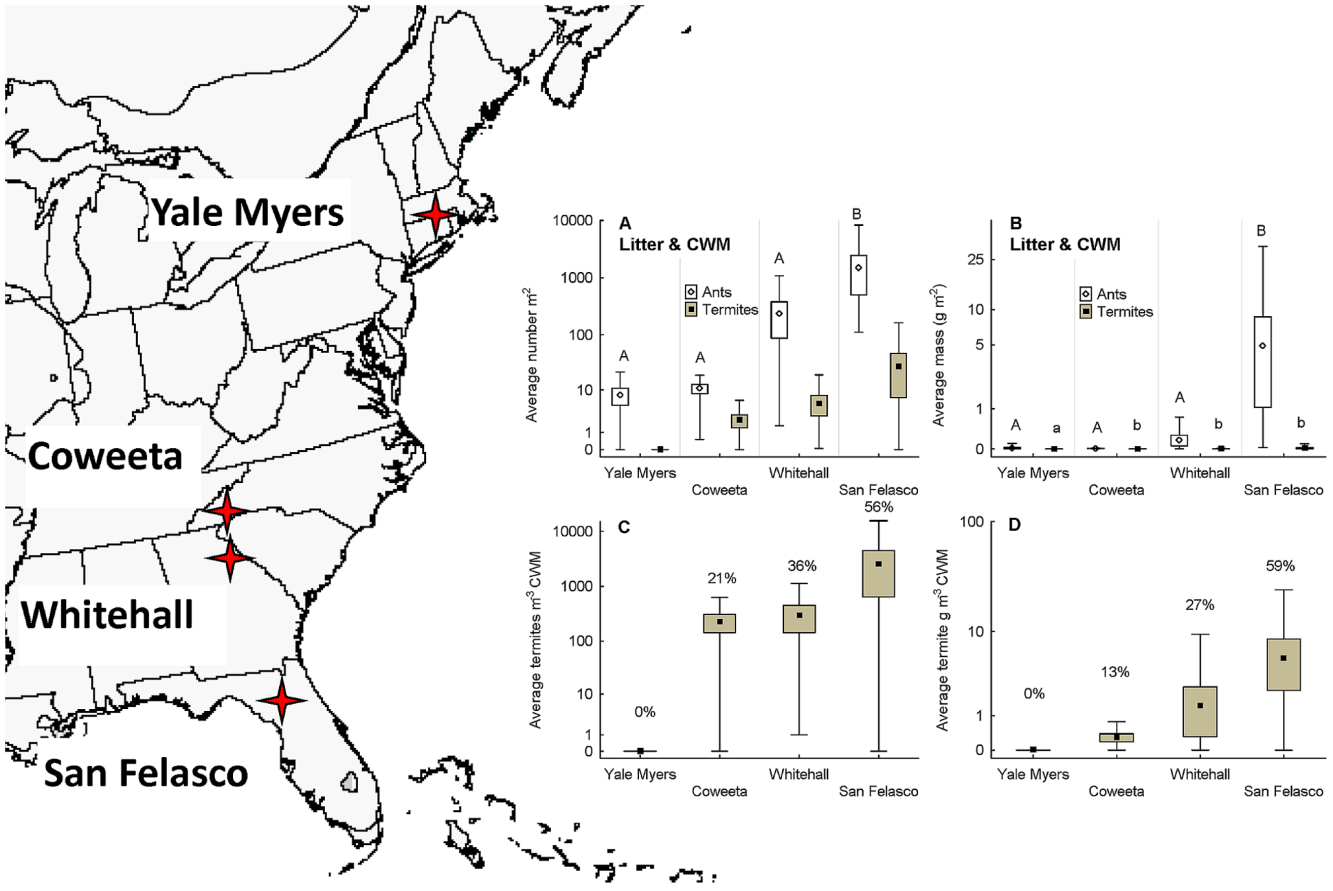
The location of sample sites and the average abundance m^−2^ (A) and g dry biomass m^−2^ (B) of ants and termites in combined litter and CWM samples. San Felasco (Florida) had a much greater abundance and biomass of ants than other sites, while termites did not vary in abundance. (C) The average g dry mass of termites m^−3^ and (D) the average number of termites m^−3^ in coarse woody material (CWM) in plots. Termite dry mass and numbers were zero at Yale Myers and did not differ significantly among the other sites. Points = mean, bars = +/− SE, and whiskers = range. The Y-axis is log_10_ scaled. Percentages above whiskers in (C and D) represent the mean proportion of invertebrate numbers and biomass in CWM that termites comprised. Map image derived from http://upload.wikimedia.org/wikipedia/commons/d/de/Eastern_US_range_map_blank.png, created by Alan Rockefeller.

When litter and CWM samples were pooled, it was apparent that non-social invertebrate biomass did not differ markedly across locations, and its abundance was greatest in Florida (San Felasco) and least in Georgia (Whitehall) (Fig. 2E, F). The average dry mass of non-social invertebrates m^−2^ was only slightly less than 1 g in the two northernmost sites [0.94 g in Connecticut (Yale Myers), 0.98 g in North Carolina (Coweeta)], whereas it was 0.25 g and 0.69 g in Georgia and Florida, respectively (Fig 2F). In contrast, there were pronounced effects of site on social insect abundance and biomass (Fig. 2E, F), which increased as latitude decreased, resulting in the highest values for Florida, especially for ants (Fig. 3A, B). Termite abundance also increased with decreasing latitude but to less of an extent, and biomass varied only slightly across locations (Fig. 3A, B).

In Florida (San Felasco), average abundance of ants (Fig. 3A) was nearly ten times greater than that of other invertebrates (Fig. 2E) and average biomass more than ten times greater (Figs. 3B and 2F). Ant mass and abundance were, on average, a minimum of five times greater than for termites in all sites and peaked at nearly 500 times (dry mass) and 55 times (abundance) greater in Florida (Fig. 3B). In all sites except Connecticut (Yale Myers), where termites were not found (Fig. 3), there were more ant than termite species (Table 1).

Though we did not observe termites in Connecticut, *Reticulitermes flavipes* has long been known to be present in the state, but uncommon [39], [40], and has been observed at the Yale Myers site (MAB, pers. obs.). In litter across the other sites, the relative abundance and biomass of termites m^−2^ was very low (average of <1%, Fig. 3A, B) whereas ants accounted for the vast majority of social insect biomass (Fig. 3B). In contrast, except for Connecticut, termites were a major component of the abundance and biomass of macroinvertebrates in CWM (Fig. 3C, D). Thus, measuring termites m^−3^ CWM likely provides the most accurate estimate of standing termite biomass in eastern US temperate forests. Ant colonies also were abundant and, combined with termites, made up a large majority of the abundance of invertebrates in CWM (Yale Myers: average ant abundance = 63%, average termite abundance = 0%; Coweeta: ants = 71%, termites = 21%, Whitehall: ants = 63%, termites = 36%; San Felasco: ants = 30%, termites = 56%; Table 3).

**Table 3:** The global reported ranges of numbers of individuals m^−2^ and biomass m^−2^ for ecosystem engineers and macroinvertebrates.

Source	Ants m^−2^/g m^−2^	Termitesm^−2^/g m^−2^ ***	Earthwormsm^−2^/g m^−2^ ***	Othermacroinvertebratesm^−2^/g m^−2^ ***	Ants%/Termites %(maximum)**
This study					
Yale Myers (41° N)	0–22/0–0.102	0/0	0–3/0–0.300	18–83/0.108–4.003	2.5%/0%
Coweeta (35° N)	1–19/0.001–0.018	0–6/0–0.005	0/0	10–47/0.098–5.186	0.3%/0.09%
Whitehall (33° N)	2–1084/0.003–0.739	1–19/0–0.013	0/0	5–23/0.079–0.823	47%/0.8%
San Felasco (29° N)	111–8310/0.027–31.578	0–163/0–0.091	0/0	45–268/0.185–1.506	95%/0.3%
[68] Bignell & Eggleton					
Tropical forests (Africa, Asia, Neotropics)	NA	38–6957/0–33.264	NA	NA	NA
Tropical savannas (Africa)	NA	49–4402/0.216–2.990	NA	NA	NA
Temperate forests (Australia)	NA	NA/0.810–1.350	NA	NA	NA
Temperate scrub and grasslands (Australia, USA)	NA	NA/0.262–1.350	NA	NA	NA
[12] Wood & Sands					
Temperate forest (Australia)	NA	600/0.810	NA	NA	NA
Semi arid savanna and grasslands (North America, Africa)	NA	0–9127/0–5.997	NA	NA	NA
Tropical savannas (Africa, Australia)	NA	70–4402/0.459–2.997	NA	NA	NA
Tropical Forests (Africa, Southeast Asia, Neotropics)	NA	87–4450/0.027–2.970	NA	NA	NA
[69] Baroni-Urbani & Pisarski					
Various (mostly temperate Europe and USA)	0–115,825/NA	NA	NA	NA	NA
[21] Kaspari & Weiser					
Various (New Worldtemperate to tropics)	NA/<0.010–<1.000	NA	NA	NA	NA
[9] Lavelle & Spain					
Various (worldwide “cold,” temperate, and tropical)	NA	NA	∼20–120/∼0.6–∼ 24.3	NA	NA
[14] Lavelle					
Tropical grasslands(Ivory Coast, Mexico)	500–1400/0.273–0.525	2–1200/<0.100–0.756	230–700/3.345–7.350	147–558/0.240–14.370	0.9%/1.4%
[37] Callaham & Hendrix					
Appalachian Piedmont(33° N, USA)	NA	NA	0–120/0–∼ 8.250 *	NA	NA
[70] Shakir & Dindal					
Various temperate forests (43°N, USA)	NA	NA	37–200/0.375–4.785*	NA	NA
[71] Suarez et al.					
Temperate hardwood forest (42°N, USA)	NA	NA	22–99/0.9660–8.085 *	NA	NA
[72] Hendrix et al.					
Southeastern pine forest(30°N, USA)	NA	NA	2/0.900	NA	NA
[15] Petersen & Luxton					
Tundra	0/0	0/0	NA/0.330	NA/0.550	0%/0%
Temperate grasslands	NA/0.1	0/0	NA/3.100	NA/1.410	2%/0%
Tropical grasslands	NA/0.3	NA/1.000	NA/0.170	NA/0.075	19%/64%
Temperate coniferous forests	NA/0.01	0/0	NA/0.450	NA/0.570	1%/0%
Temperate deciduous forests	NA/0.01	0/0	NA/0.200–5.300	NA/1.280	0.2–0.6%/0%
Tropical forests	NA/0.03	NA/1.000	NA/0.340	NA/0.060	2%/70%

* Majority exotic species.

** Percent of maximum biomass (all macroinvertebrates) reported.

*** Conversion of fresh weights to dry weights (g) are estimates and followed that of [15]: termite fresh weight×0.27 = dry mass, earthworm fresh mass×0.15, ant fresh mass×0.23, and other macroinvertebrates fresh mass×0.30. These conversions do not apply to the invertebrates sampled in this study as those were dried and weighed.

The number of social insect colonies m^−2^ collected from litter and CWM were higher in southern sites, especially Florida (extrapolations for Yale Myers and Coweeta: ∼2 colonies m^−2^, Whitehall: ∼5 colonies m^−2^, San Felasco: ∼13 colonies m^−2^). A majority (65%) of ant colonies collected included queens (were queenright) and colony size numbers (i.e. for non-queenright colony fragments) produced from this sampling method generally match other published estimates for these species [41], [42]. A few species were, by far, the most abundant both in terms of total abundance (numbers of individuals) and colony abundance, comprising greater than 50% of ant or termite workers in all sites (Table 4). Termites were almost entirely *R. flavipes*, although one colony of *R. hageni* was collected in Florida (Table 4 and Table 1).

**Table 4:** The abundance of the most common species of ants and termites collected in each site.

Social insect	Site	Species	Number of colonies or occurrences		Average workernumber		% of total abundance
Ants	Yale Myers	*Aphaenogaster picea*		10		414	100
Termites	Yale Myers	NA		0		0	0
Ants	Coweeta	*A. picea*		20		189	64
Termites	Coweeta	*Reticulitermes flavipes*		11		161	100
Ants	Whitehall	*P. dentata*		7		692	68
Termites	Whitehall	*R. flavipes*		13		181	100
Ants	San Felasco	*Camponotus floridanus*		2		2727	55
Termites	San Felasco	*R. flavipes*		12		1684	98

Colony numbers (ants) and occurrences in CWM (termites) as well as average number of workers are shown. Percent of total abundance was determined for ants and termites separately as a percentage of the total number of workers captured.

A broad-scale pattern emerged among the most common species whereby *Aphaenogaster picea* was, by far, the most dominant ant in the two northernmost sites (Table 4). In Georgia (Whitehall), however, *Pheidole dentata* was the most common ant species (although a closely related *A. picea* congener, *A. rudis,* still was present in considerable numbers). *Pheidole dentata* had the most colonies in Florida, too (although the carpenter ant *Camponotus floridanus* had a higher number of workers and biomass in Florida due to sampling two large colonies – Table 4). The genus *Aphaenogaster* was not found in the Florida site although it is present in San Felasco State Park [43], whereas the species *A. rudis* and *A. picea* are not present in Florida [44]. The abundance of ants in CWM results from the predominance of *Aphaenogaster* and *P. dentata* colonies (Fig. 3, Table 4). These species opportunistically nest in rotting wood and will typically have portions of the nest extending into the soil (JRK, RJW, MAB pers. obs.). The highest diversity of ant species occurred in North Carolina (Coweeta) and Georgia (Whitehall), each with ten species (Table 1). These sites also had five species in common (Table 1).

Average soil temperature at the time of sampling differed among sites (*ANOVA, P*<0.0001), as did soil moisture (*ANOVA, P*<0.0001). Connecticut (Yale Myers) was, on average, the coolest and wettest site (18.4°C, 13.2% soil moisture) and North Carolina (Coweeta) was the warmest and driest (24.6°C, 3.2% soil moisture). Georgia (Whitehall) and Florida (San Felasco) were similar (22.8°C, 9.6% soil moisture; 23.1°C, 5.6% soil moisture, respectively). Only termite abundance had a linear relationship with soil temperature across plots (ant abundance *P* = 0.25, *R* ^2^ = 0.04; termite abundance *P* = 0.02, *R* ^2^ = 0.17; invertebrate abundance *P* = 0.55, *R* ^2^ = 0.01) although it was weakly correlated. There was no relationship between soil moisture and abundance for any group (ant abundance *P* = 0.38, *R* ^2^ = 0.03; termite abundance *P* = 0.21, *R* ^2^ = 0.05; invertebrate abundance *P* = 0.36, *R* ^2^ = 0.03).

Both annual temperature and productivity show that our sites are representative of a latitudinal gradient where temperature and productivity increased with decreasing latitude. Using San Felasco Hammock (the southernmost and annually warmest site) as a baseline “0,” assignment of general elevational and latitudinal lapse rates (−6.5°C per 1000 m increase in elevation and −1°C per 145 km north increase in latitude), showed that Whitehall Forest was annually 3.75°C cooler, Coweeta was 9.21°C cooler, and Yale Myers was 10.86°C cooler than the Florida site. Thus our sites comprise a climate gradient where temperature declined with latitude and Coweeta and Yale Myers sites (North Carolina and Connecticut) group as northern sites that were closer in annual temperature than the southern sites at Whitehall and San Felasco (Georgia and Florida). Above ground annual production by plants in each site was estimated as 1500 g m^−2^ yr^−1^ at San Felasco Hammock, 1050 g m^−2^ yr^−1^ at Whitehall Forest, 920 g m^−2^ yr^−1^ at Coweeta, and 840 g m^−2^ yr^−1^ at Yale Myers Forest.

## Discussion

The relative abundance of ants in the southern temperate sites was unexpected and is comparable to the impressively high abundance and biomass of ants in tropical arboreal ecosystems (up to 70% of all arboreal arthropods, up to 50% of arboreal arthropod biomass; [45], [46], [47]) and higher than estimates for tropical ground-dwelling ants (e.g. [21], [48]). Furthermore, our average areal biomass estimate (4.87 g dry mass m^−2^)in Florida (San Felasco) equals or surpasses most other commonly abundant, terrestrial vertebrate, animal groups such as salamanders in northeastern temperate forest in the US (∼0.05 g dry mass m^−2^), British Virgin Island reptile communities (∼0.0001 g dry mass m^−2^), all large (greater than 500 g body weight, including elephants) mammals in equatorial rainforest in Gabon (∼0.32 g dry mass m^−2^), mammals in dry tropical forest in Thailand (0.73 g dry biomass m^−2^), mammals in a variety of neotropical forest and grassland (0.11–0.33 g dry biomass m^−2^) and even pastures stocked with cattle (up to 2.28 g dry biomass m^−2^) in Brazil (approximation of dry mass values = 0.3×fresh mass values reported in [23], [49], [50], [51], [52]).

In contrast to the patterns observed in the southern sites, termites and ants were subordinate within the macroinvertebrate communities in the northern part of the range of eastern temperate forests (Fig. 2E, F). This result supports the long-standing consensus that ants are thermophilic (warm-loving) and their abundance is greater in ecosystems with higher primary productivity, especially where temperatures are higher [1], [21], [26], [27]. Termites are also a thermophilic taxon and their abundance is greater in warmer regions [2]. Our data (Figs. 3C and D) and those of Vargo et al. [53] show a large increase in termite abundance in warmer regions within temperate zones. Temperate termite abundance may also be affected by the availability of standing and downed dead woody material (Fig. 3C, D), however, the more northern part of the temperate zone has higher CWM stocks than in the south [54], again suggesting that cooler climate is an important limit on their abundance (Fig. 3) [53], [55], [56].

An interesting example of biogeographic turnover occurred in the most abundant ant species in CWM from northern (*A. picea*) to southern (*P. dentata*) sites (Table 4). These two species are ecologically similar despite being in different genera. Both species opportunistically nest in decaying wood and soil, have very similar diets (both prey upon termites and both will take eliasome bearing seeds), appear to be weakly territorial or not territorial at all, and have colonies that are typically below 1,000 workers in size (JRK, RJW, MAB pers. obs.). The divergence in the ant communities that occurs somewhere between North Carolina and Georgia is almost certainly under climatic influence, with the cooler temperate species (*A. picea*), giving way to the southeastern coastal plain species (*P. dentata*). Termites showed no such pattern, with *R. flavipes* remaining the dominant species throughout the entire range of the study (Table 4), but termite biomass in CWM does increase markedly between North Carolina and Georgia.

Variation in sampling protocols for soil fauna complicates the comparison of our results with other studies (i.e. Table 3). We under-sampled earthworms in our study, and possibly other groups (e.g. fast-moving large spiders), although the remaining macroinvertebrate fauna are well-represented [15]. Our sampling approach thus appears useful for estimating areal abundance of social insects and most co-occurring macroinvertebrates. Notably though, our sampling design was likely effective at estimating areal biomass but not effective at capturing whole colonies of termites. This is because termites in the genus *Reticulitermes* are dead wood-feeding species of the “multiple-piece nesting” functional group, which means that they feed upon decaying wood away from the primary nest where the reproductive members of the colony reside [57], [58]. Nests are cryptic and the majority of above and belowground termite abundance represents feeding rather than nesting activity (nests include sexuals and some workers), though nests sometimes are located above ground [59]. Colonies tend to be simple family groups, comprising a single reproductive pair (a queen and king) and their offspring, and maintain foraging areas typically ≤100 m^2^ in size in the southeastern US [53]. Inbreeding, larger territories, and extended family colonies become more common in the northern part of the range where abundance and colony density is also lower (Figs. 3C and D, [53]).

In contrast to termites, and due to the fact that the majority of the ant colonies collected were queenright (65%), monogyne (single queen), monodomous (single nest) species (Table 1), our sampling protocol appears to be effective at estimating both areal biomass and colony abundance of ground-dwelling ants. Despite the high numbers and biomass we report, it is important to note that our areal abundances are still underestimates. At Yale Myers, Coweeta, and Whitehall we also searched for ants under rocks and at Yale Myers in fine woody material (FWM, <10 cm dia.). Using Yale Myers as an example, in addition to the 10 *A. picea* colonies in CWM we used for this analysis, we did not include seven colonies found under rocks and fourteen colonies found in FWM, which contained the only colonies with >1,000 workers. If this under-sampling holds across all our sites, then the true biomass and numbers of ants may be ∼3-times larger than the already high values we report, with the obvious caveat that these are extrapolations. We did under-sample ant species diversity because of the relatively small number of samples and use of one, rather than multiple, sampling techniques [60], [61].

Maintaining ecosystem services is a critically important, central component of global biodiversity conservation strategies [62]. Lavelle [5], [7], [9], [63] and others [2], [5], [8], [10], [16] have called attention to the central importance of social insects, along with earthworms, in maintaining soil ecosystem function and all of the associated ecosystem services. If the importance of social insects for soil processes is at least partially dependent upon biomass [12], then the data we present here suggest ants and termites are among the most important macroinvertebrates in eastern US temperate forests, at least in the southern parts of the range and likely in other temperate systems (Table 3).

**Figure 4:**
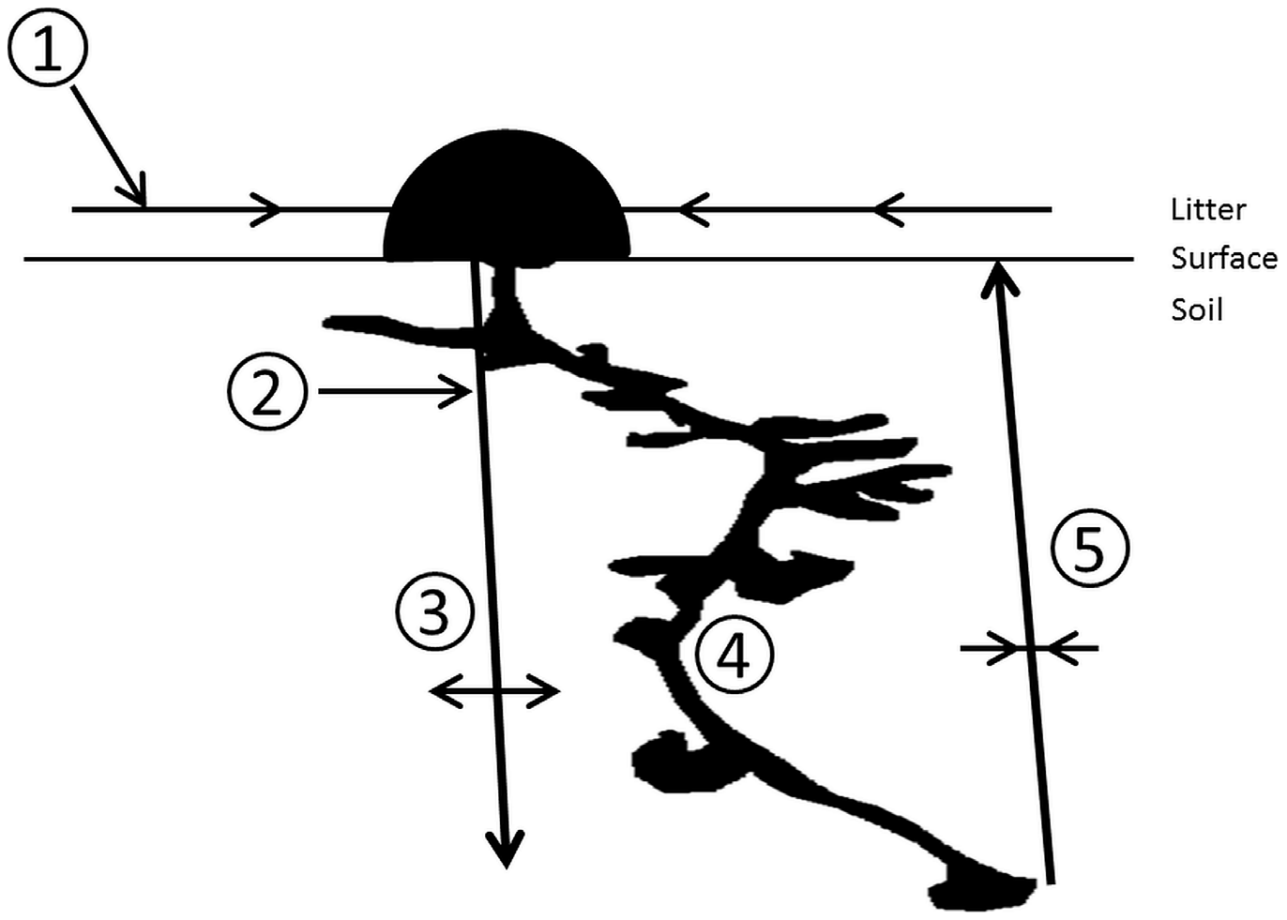
The myrmecosphere is centered upon ant nests constructed at the soil surface and below ground. (1) Prey and carrion, plant material, plant and insect exudates are brought into the colony. (2) Below-ground prey and carrion, plant material, plant and animal exudates are brought into the colony. (3) Materials brought into the colony are assimilated into the soil over time. (4) Feces, saliva, and other excretions are produced within the colony. (5) Soil, corpses, and midden material are returned to the soil surface.

Lavelle et al. [64] identify four principal systems of biological regulation of decomposition and soil structure: the litter-superficial root system, the rhizosphere, the drilosphere, and the termitosphere. The drilosphere and the termitosphere are the processes under the influence of earthworm populations and termite populations, respectively, through their activities in the soil environment. These include intestinal contents, castings, and galleries for earthworms and mounds, galleries, woody material, and gut symbionts for termites. No such system of biological regulation has been identified for ants: a myrmecosphere, Given the huge abundance and biomass of ants we observed in the southern part of our system, the myrmecosphere might be considered a fifth system of biological regulation in soils. It’s contribution to the soil ecosystem, particularly the physical structure and chemical make-up of the soil environment [5], [10], [65], would be an emergent property of the social organization of colonies and the nests they construct and maintain [66]. The nest is the organizational centerpiece of colonial living for ants, shaping the spatial arrangement of individuals and division of labor [67] as well as the movement of materials into and out of the colony (Fig. 4). The nest is thus the “building block” of the myrmecosphere (Fig. 4). More data on areal abundance (Table 3) and the belowground activities of ants [66], [67] are necessary to better quantify the functional role of a myrmecosphere in ecosystems.

The paucity of data on ant and termite abundances is cited as the reason for omitting them from syntheses of biogeographical patterns in belowground communities [17]. Our observations begin to redress this shortcoming and reveal a pronounced shift from social insect subordinance to dominance across decreasing latitude in a major, temperate forest ecosystem. Termites were the most abundant macroinvertebrates in dead wood and ants were the most abundant in litter and soil. Ant abundance and biomass m^−2^ in the southernmost site were among the highest values recorded for ants in any ecosystem, highlighting the potential importance of these faunal groups to the belowground functioning of temperate systems.
