# Correction: Social Insects Dominate Eastern US Temperate Hardwood Forest Macroinvertebrate Communities in Warmer Regions

**DOI:** 10.1371/annotation/87285c86-f1df-4f8b-bc08-d64643d351f4

**Published:** 2013-10-22

**Authors:** Joshua R. King, Robert J. Warren, Mark A. Bradford

This article has been republished to replace Figure 3 due to copyright issues. 

